# Novel active-feedback device improves sleep quality in insomnia disorder: a randomized placebo-controlled trial

**DOI:** 10.3389/frsle.2025.1452213

**Published:** 2025-05-16

**Authors:** Antonia Ypsilanti, Maan van de Werken, Anna Robson, Petra Examilioti, Lambros Lazuras

**Affiliations:** ^1^Department of Psychology, Sociology, & Politics, Sheffield Hallam University, Sheffield, United Kingdom; ^2^Braintrain2020 Limited, Sheffield, United Kingdom; ^3^Department of Sports Sciences, Lincoln University, Lincoln, United Kingdom

**Keywords:** sleep, insomnia, treatment, insomnia severity index, consensus sleep diary, sleep efficiency

## Abstract

**Objectives:**

Insomnia disorder is a public health challenge associated with impaired cognitive functioning, reduced quality of life, and adverse mental health outcomes. This study examined the effectiveness of SleepCogni, an active-feedback device, in reducing insomnia symptom severity and improving perceived sleep quality in individuals with insomnia disorder.

**Method:**

A two-arm, parallel-group trial design was used, with 80 participants randomized to either the experimental group or the placebo control group. Participants provided self-reported measures of insomnia severity, sleep continuity, and user experience as main outcome variables.

**Results:**

Repeated measures analysis of variance showed that participants in the experimental group reported significantly greater improvements in insomnia symptom severity than the control group (Time × Treatment). Although the mean difference did not reach the threshold for clinical significance, 37.5% of the participants achieved this threshold within 1 week of treatment. Mixed-effects models showed significant improvements in sleep efficiency and total sleep time, based on sleep diary records. Finally, an independent samples *t*-test and content analysis indicated a more positive user experience in the experimental group.

**Conclusion:**

The SleepCogni device appears to be a useful tool for improving sleep outcomes in individuals with insomnia disorder, showing effects on both insomnia severity and subjectively evaluated sleep. The SleepCogni device presents a useful intervention that might be used alone or as a complement to increase the effectiveness of existing treatments, such as cognitive behavioral therapy for insomnia.

**Clinical trial registration:**

https://osf.io/rswcb, identifier: osf-registrations-rswcb-v1.

## Introduction

Insomnia is a sleep disorder characterized by difficulties in initiating and/or maintaining sleep and is associated with impairments in daytime functioning, such as fatigue, sleepiness, and impaired cognitive performance (Riemann et al., 2017). Epidemiological studies estimate insomnia's prevalence to be between 10% and 13.5% in both adolescents and adults (Johnson et al., 2006; Sivertsen et al., 2009). Furthermore, research has shown the prevalence of insomnia increased significantly (37.6%−38.9%) during the COVID-19 pandemic (Pappa et al., 2020; Voitsidis et al., 2020). Insufficient sleep among people with insomnia symptoms has been associated with an increased risk for myocardial infarction, stroke, depression, and anxiety (Kalmbach et al., 2016), and insomnia increases the risk for anxiety, depression, and alcohol abuse (Hertenstein et al., 2019).

Treatment options for insomnia are limited, with cognitive behavioral therapy for insomnia (CBT-I) being the first-line treatment. CBT-I is considered more effective and safer than pharmacotherapy (Buscemi et al., 2007; Riemann et al., 2017). Reviews and meta-analyses show that CBT-I interventions yield moderate-to-large short-term effect sizes in the short term with improved sleep efficiency (the ratio between total sleep time and total time planned for sleep) and reduced severity of insomnia symptoms (Soh et al., 2020; Trauer et al., 2015; Van Straten et al., 2018). Meta-analyses have also shown that moderate to large effect sizes were maintained at follow-up. However, people suffering from insomnia have only limited access to CBT-I largely due to the limited availability of CBT-I therapists (Hertenstein et al., 2022; Koffel et al., 2018; Simon et al., 2023). The current study tests a handheld device that can support CBT-I.

**Figure 1:**
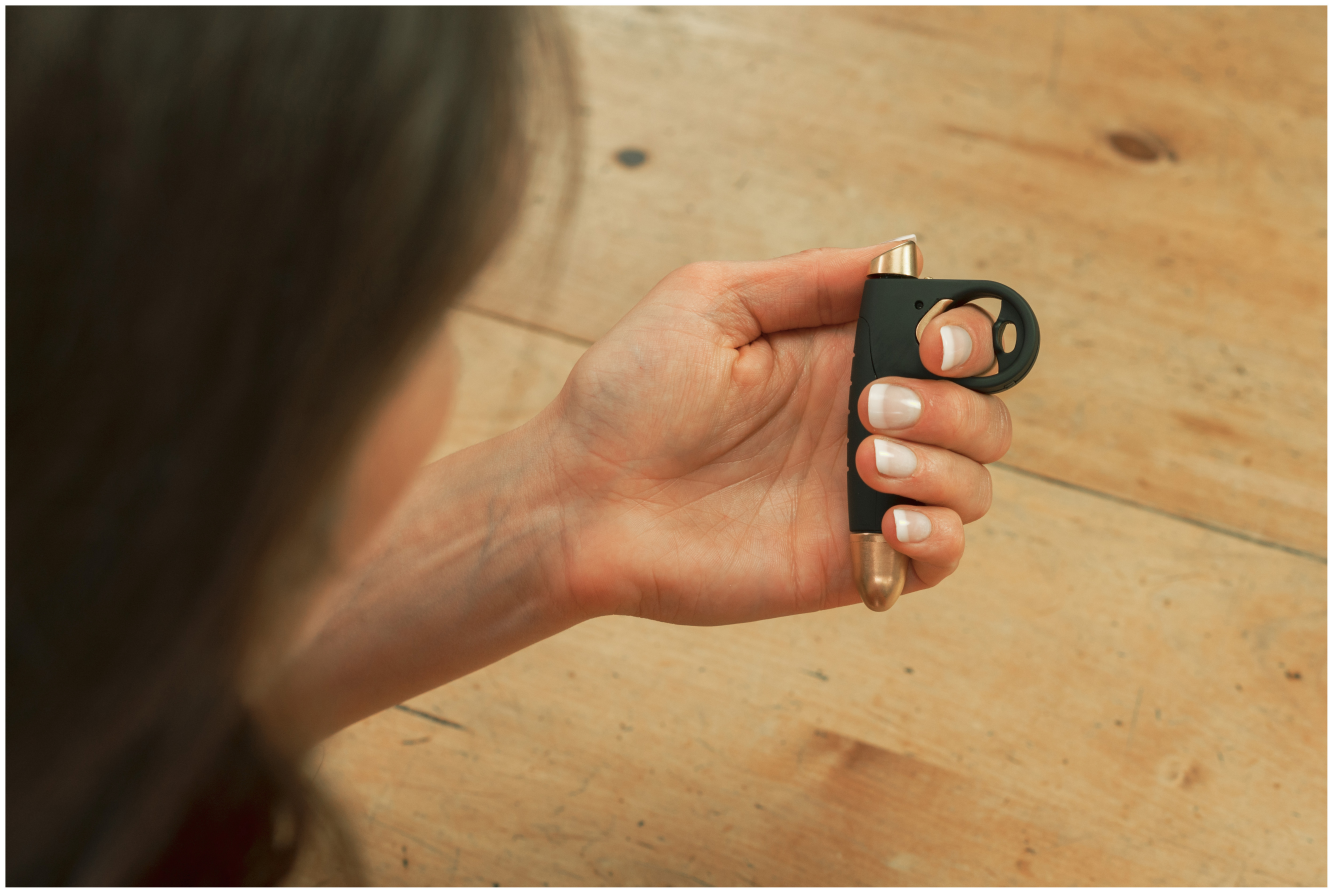
Photograph of the SleepCogni handheld device. Tactile stimuli cues are provided to the user's hand. User input is received when pressing the button on top of the device in response to each cue. Physiological sensors are present in the ring.

Although digitalized CBT-I (online platforms) has recently been developed and investigated (Espie et al., 2019; Stenberg et al., 2022), it tends to lack critical user engagement compared with therapist-led interventions (Fairburn and Murphy, 2015; Van Ballegooijen et al., 2014), and considerable dropout has been observed (Zachariae et al., 2016). Furthermore, CBT-I does not provide the user with direct, on-demand support at the time and place when the need for support is greatest, typically during sleep onset or sleep reinitiation. Insomnia is often recurrent, and long-term treatment is required. Therefore, securing and maintaining adherence to treatment for insomnia could be critical for long-term efficacy (Mellor et al., 2022). Handheld technology that provides support during sleep initiation or reinitiation can fill this gap in treatment options for people suffering from insomnia (Figure 1).

This study was a randomized placebo-controlled trial aimed at assessing the effectiveness of a novel handheld technology, SleepCogni (developed by Braintrain2020 Ltd.), for treating insomnia symptoms. SleepCogni measures physiology and behavior related to sleep initiation (e.g., skin temperature and reaction times) and delivers an intervention that supports “active rest” prior to sleep initiation or reinitiation. The device provides tactile stimuli cues to the user that gradually decrease in frequency and intensity in real time and in response to the user's physiological and behavioral measures. SleepCogni's algorithm incorporates active (e.g., reaction time: the time between stimulus cue and button press) and passive (physiologic; e.g., skin temperature at the index finger) inputs from the user to generate a stepwise relaxation program that supports sleep initiation and reinitiation.

The key rationale for SleepCogni's insomnia treatment is that it supports an “active rest” state, reducing hyperarousal. To do this, SleepCogni requires the user to engage with the handheld device by acknowledging each tactile stimulus cue by pressing a button on the device. This exercise aims to support user engagement in the process of “active rest” by breaking cognitive cycles that interfere with sleep as the user's attention is shifted toward responding to tactile cues. The high frequency of tactile cues at the start of the program is intended to require such a high level of attention that no time is left for any thoughts to start wandering. When skin temperature and reaction times start to increase (a sign of increased sleepiness), the frequency and strength of these tactile cues decrease, becoming more randomly spaced and harder to predict. Engagement with the later phase of the program is intended to require sustained attention, which is expected to become harder with increasing sleepiness. SleepCogni's therapy is thus hypothesized to reduce hyperarousal and negative thoughts and behaviors (e.g., rumination and sleep effort) associated with sleep initiation and reinitiation in people suffering from insomnia by offering a replacement behavior that distracts the user from their habitual thought processes and allows them to rest, thereby engaging in sleep initiation or reinitiation. The data obtained using the SleepCogni handheld device further provide important information about timing and duration patterns in difficulties with initiating or reinitiating sleep in this population that can be used in the future to support CBT-I (not part of the current study).

This study used a randomized, placebo-controlled trial design to assess the effectiveness of the SleepCogni device in alleviating insomnia symptoms and improving sleep quality in individuals suffering from insomnia disorder. The primary outcomes included subjective insomnia severity measurements and sleep continuity indicators as reflected in subjective sleep diaries. The secondary outcomes included subjective user experience characteristics. The following was hypothesized:

H1: Participants in the experimental group will report lower scores in insomnia severity and improved subjective sleep quality compared to the control group.

H2: Participants in the experimental group will report a more positive user experience than those in the control group.

## Materials and methods

### Participants

Participants were recruited via opportunity sampling and through advertising the study in British universities in the West Midlands and the Yorkshire area. The researchers developed a volunteer list using a series of self-reported questions to which participants must have declared agreement. Exclusion criteria were night-shift workers and those taking hypnotics or other medications to induce sleep. Following this initial screening process, participants were included in the volunteer list and received further screening using a study screening questionnaire that assessed sleep quality and daily sleep routines, medical history, demographic information, and symptoms of other sleep disorders (e.g., snoring).

#### Eligibility and exclusion criteria

Participants were eligible to participate in the study if they had met the criteria for a diagnosis of insomnia disorder, consistent with the *Diagnostic and Statistical Manual of Mental Disorders, Fifth Edition* of the American Psychiatric Association (2013). Insomnia symptoms (Insomnia Severity Index [ISI] score > 8) should be present for at least 3 nights per week for at least 3 months and cause significant daytime impairment to meet the chronic insomnia criteria.

Furthermore, participants were excluded from the study if they self-reported having any of the following characteristics: suffering from a comorbid sleep disorder (e.g., sleep apnea); experiencing life circumstances that affect sleep patterns (e.g., including being pregnant, breastfeeding, and menopause); using medication known to affect sleep–wake function during the past 3 months; traveling over two (or more) time zones on transmeridian flights 30 days prior to taking part in the study; working night shifts in the week prior to the study or any other shift work during the last 3 months; having a diagnosis of psychological disorder (clinical depression, anxiety, posttraumatic stress disorder, or psychosis); having a history of chronic disease (e.g., chronic pain, fibromyalgia, cancer, chronic obstructive pulmonary disease, or osteoarthritis); suffering from Raynaud's disease; consuming excessive amounts of caffeinated drinks daily such as coffee (more than 4 cups per day); having a history of or being under treatment for problematic alcohol use (i.e., consuming more than 14 units of alcohol per week) or substance misuse; having a Body Mass Index <16 or >34; and having a pacemaker.

Following our preregistration, we aimed to recruit approximately 75 subjects, based on a power analysis, to obtain 0.95 power to detect a medium effect size of 0.25 at the standard 0.05 alpha error probability. We overshot this aim slightly and included 80 participants reporting insomnia disorder who satisfied the inclusion/exclusion criteria. Of those who participated in the study, the mean age was 26.3 (*SD* = 10.8, range: 18–72 years old), and 63 (79%) were women. Note that one subject was not included in the study as they did not use the device at all and was replaced by an additional subject following immediate recruitment. They were randomized blindly to active and sham (control) devices. The study took place between October 2020 and June 2021. Participants were compensated for their time after completing the study (see the Supplementary material for a flowchart of the trial).

### Measures

#### Insomnia severity index, primary outcome

Pre-treatment and post-treatment, the ISI was used to assess the severity of insomnia symptoms (Bastien et al., 2001). The post-treatment ISI measurement was taken the day after completing the week of treatment. This is a self-reported measure that includes seven items that assess the nature, severity, and impact of insomnia, on a 5-point continuous scale (0 = *no problem* to 4 = *very severe problem*). Scores are summed to generate a total ISI score ranging from 0 to 28, with higher scores indicating greater insomnia severity. The ISI cutoff scores can be used to determine insomnia severity: 0–7 = absence of insomnia, 8–14 = sub-threshold insomnia, 15–21 = moderate insomnia, and 22–28 = severe insomnia. The ISI indicated good internal consistency/reliability at baseline (Cronbach's α = 0.74).

#### Consensus sleep diary, primary, and secondary outcomes

During treatment, the Consensus Sleep Diary (Carney et al., 2012) was used to assess subjective sleep continuity. Participants were required to fill in the sleep diary every morning upon awakening, having used the device the night before. As secondary outcomes, the data from the diary were used to calculate sleep onset latency (SOL; in min), the number of awakenings after initial sleep onset, wakefulness after sleep onset (WASO; in min), and early morning awakening (EMA; in min). Total sleep time (TST; in min) was calculated as the duration between sleep onset and final awakening minus WASO. As a primary outcome, sleep efficiency (SE; in %) was calculated as the total sleep time across the duration of the sleep episode from lights out to intended wake time (Reed and Sacco, 2016). In addition, the sleep diary offered volunteers the opportunity to leave voluntary comments about their night's sleep (secondary outcome).

#### User experience questionnaire, secondary outcomes

At the end of the trial, participants completed a user experience questionnaire. User experience reflected perceived usefulness (5 items; e.g., “Using the device improved my effort to fall asleep,” “Using the device reduced my worries over falling asleep”; Cronbach's α = 0.84), perceived easiness of using the device (2 items; e.g., “Overall, I found it easy to use the device,” “Learning to use the device was easy and understandable”; Cronbach's α = 0.70), perceived easiness of falling asleep using the device (2 items; e.g., “It was easy to fall asleep with the device,” “I did not face any difficulties falling asleep with the device”; Cronbach's α = 0.74), intentions to use the device in the future (1 item, “If you had the opportunity, how likely would you be to use the device in the future?”) intentions to recommend the device to others (1 item, “Would you recommend the device to a friend or relative of yours who faced sleep problems?”), and overall satisfaction with using the device (1 item, “Overall, how satisfied are you with using the device”).

Responses in perceived usefulness and ease of using the device were recorded on a 5-point Likert scale (1 = *Strongly Disagree*, 5 = *Strongly Agree*). Responses to intentions to use the device, recommend it to others, and overall satisfaction were recorded on a continuous scale (0 = *not at all*, 10 = *very much*). Higher scores in all measures reflected higher levels on the corresponding variable. Participants were further asked to report whether using the SleepCogni (or the sham) device to fall asleep was more effective, equally effective, or less effective than other approaches or treatments for insomnia they used in the past. See the Supplementary material for the full user experience questionnaire used in the study.

#### Demographic and baseline measures

Regarding demographic characteristics, participants declared their age (open-ended question) and their biological sex (male or female) at the beginning of the questionnaire at baseline. Additionally, participants completed the Hospital Anxiety and Depression Scale (HADS; Zigmond and Snaith, 1983) as a baseline measure of anxiety and depression symptoms. Note this questionnaire was not used to exclude subjects, for which we only used self-reported clinical diagnoses. The HADS includes 7 items for anxiety and 7 items for depression scored on a 4-point continuous scale ranging from 0 to 3, with a maximum score of 21. Higher scores indicate higher anxiety and depression. Both subscales demonstrate good internal consistency/reliability at baseline (HADS – depression Cronbach's α = 0.83; HADS – anxiety Cronbach's α = 0.78). Baseline measurements for all metrics of both arms are provided in Supplementary Table S1.

### Design and procedure

A randomized parallel-arm design was used. Participants with insomnia who met the inclusion/exclusion criteria were randomly and blindly allocated to the experimental and placebo control groups. The study was preregistered with the Open Science Foundation and received Ethics approval from the respective Research Ethics Committee (ER20811882) of the host academic institution. All participants were blind to the condition they were assigned to as a safeguard against subject-expectancy effects. Participants in each group used the devices they were given (active device vs. sham device) for 7 consecutive nights. The devices were posted to the participants' houses (because of social distancing measures at the time of data collection) and returned to the researcher upon the study's completion via courier service.

All participants received a video explaining in detail how to use the device. They were also sent a sleep diary to complete in the morning after each night and were sent reminders to do so on their phone. The control group received the same device as the experimental group, with the only difference being that the sham device did not send tactile signals to the hand and the response button was removed. All participants were asked to use the handheld device every night at their initial sleep initiation and either respond to the tactile stimuli cues (experimental group) or just hold it (control group). SleepCogni works by providing users with random tactile cues in the handheld device to which the user responds. The frequency of these cues decreases from 30 to 8 per minute in a logistic decay function. Furthermore, the vibration intensity of the tactile cues decreases together with frequency. The decay function of the cues the user receives accelerates when a user is estimated as sleepier as determined from physiological and behavioral measures. The exact function and parameters of these user interactions are proprietary. The program is paused when the user does not respond to five consecutive cues, as this is when a user is deemed to have fallen asleep. Should a user not be asleep, a press on the handheld device resumes the program.

Participants were free to decide to use the device throughout the rest of the night to reinitiate sleep if needed. To make the control group members believe a treatment was in operation, they were told that the device delivered “homeostatic regulation” to improve their sleep without further explanation. At the end of the study, all participants were fully debriefed about the study.

### Data analysis

Insomnia severity was analyzed using repeated measures analysis of variance (ANOVA) with TIME (pre, post) as the repeated measures factor and GROUP (experimental, control) as the between-groups factor. User experience responses were analyzed using independent samples *t*-test. The effectiveness ratings of the SleepCogni device compared to self-reported previously used approaches and treatments were analyzed with Pearson's chi-square (χ^2^). Sleep diary measurements were analyzed using mixed effects models in *lmer* in R. Mixed models are flexible and can handle missing values (Bates et al., 2015). As measurements were taken across 7 nights and some data were missing, these models are preferred over repeated measures ANOVA. Missing data were due to either subjects not entering data or subjects entering data that were incompatible with each other, for example, WASO being longer than the duration from sleep onset to final awakening. When data could not be corrected, this sleep diary data entry was excluded. However, some subjects missed different nights than others and, on rare occasions, skipped a night of using the device. Chronological ordering of the data on a night-by-night resolution was therefore not possible, and we thus analyzed this as a two-level factor, indicating whether data were from the first two nights of the study or the subsequent nights (i.e., “time” in the experiment). Only subjects for whom data were available for 5 nights or more were included in the analysis for each variable. The interaction with time of the treatment variable to detect differential changes over time was examined. For each variable of interest, the full model is reported and provides the overall estimates and *p*-values obtained from *t*-tests using the *lmerTest* package in R. All data were log10(*x* + 1) transformed apart from EMA and sleep efficiency.

## Results

### Effects of the SleepCogni device on insomnia severity

**Figure 2:**
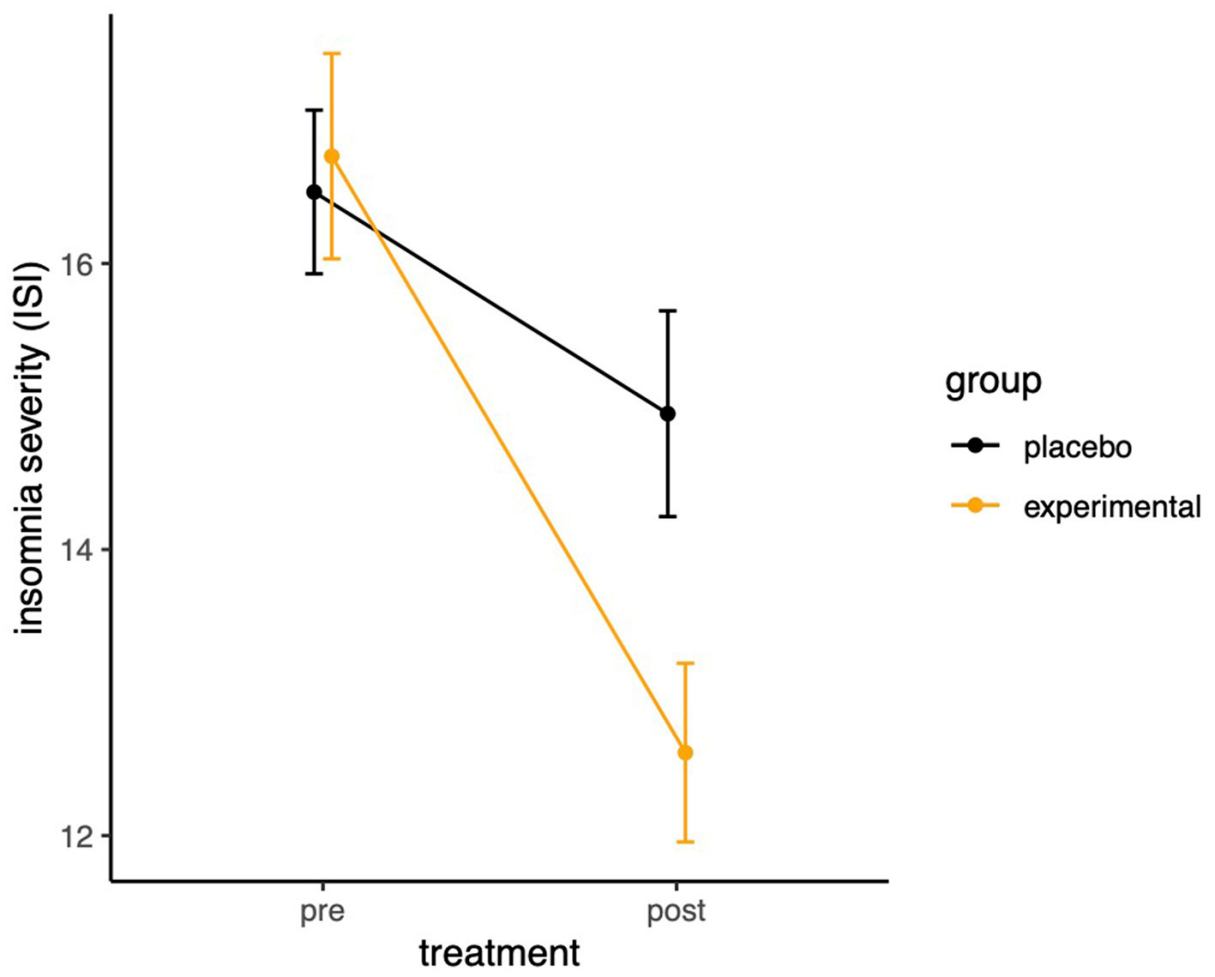
Interaction between time and experimental group allocation on insomnia severity.

Insomnia severity (using the ISI) measured before and after participating in the trial was lower in response to the treatment (Figure 2). On average, a 4.2-point reduction in ISI scores was seen in the experimental group compared to a 1.6-point reduction in the control group (Experimental group pre: *M* = 16.75, *SD* = 4.54; post: *M* = 12.58, *SD* = 3.95; control pre: *M* = 16.50, *SD* = 3.62; post: *M* = 14.95, *SD* = 4.55). There was a significant main effect of TIME (pre–post), *F*_(1,78)_ = 48.25, *p* < 0.001, $\eta_{\text{p}}^{2}$ = 0.38, and a significant interaction between TIME × GROUP, *F*_(1,78)_ = 10.14, *p* < 0.01, $\eta_{\text{p}}^{2}$ = 0.11. Note that this mean difference does not reach the 6-point clinical meaningful improvement threshold for this index (Yang et al., 2009).

### Effects of the SleepCogni device on subjective sleep continuity

The majority of the sleep diary measurements taken during the duration (but not at baseline) of the study indicated improvements in subjective sleep continuity. Specifically, SOL, the number of awakenings, WASO, and EMA decreased significantly, and SE increased significantly over time for both the experimental and control groups taken together (Time column, Table 1). In the experimental group, both TST and SE increased significantly over time compared to the control group (Time × Treatment column, Table 1). TST increased by 15.1 min in the control group compared to 56.4 min in the experimental group. SE increased by 5.4 in the control, compared to 10.7 in the experimental group.

**Table 1:** Coefficients and their standard errors from mixed effects models for the sleep diary outcomes.

**Measure**	**Intercept (control)**	**Time**	**Treatment**	**Time × treatment**	***n***
Sleep onset latency (min)	1.64 ± 0.05	−0.148 ± 0.038^*^	−0.049 ± 0.070	−0.054 ± 0.053	77
Number of awakenings after initial sleep onset	0.479 ± 0.034	−0.177 ± 0.026^*^	0.0422 ± 0.0483	0.054 ± 0.037	78
Wakefulness after sleep onset (min)	1.29 ± 0.091	−0.387 ± 0.073^*^	0.183 ± 0.130	0.017 ± 0.105	76
Early morning awakening (min)	40.4 ± 5.29	−19.7 ± 5.65^*^	−9.55 ± 7.46	8.62 ± 7.97	78
Total sleep time (min)	2.60 ± 0.017	0.016 ± 0.015	−0.058 ± 0.025^*^	0.049 ± 0.021^*^	76
Sleep efficiency (%)	78.5 ± 1.84	5.44 ± 1.51^*^	−5.78 ± 2.65^*^	5.23 ± 2.17^*^	75

Estimates are provided compared to the control group; time indicates the effect over time within the trial; the treatment effect and the interaction indicate the change over time within the experimental group compared to the control. ^*^p < 0.05.

### User experience evaluated after trial participation

Independent samples *t*-test showed that participants in the experimental group reported significantly higher scores than participants in the control group regarding the perceived usefulness of the SleepCogni device, *t*_(78)_ = 2.30, *p* < 0.05, Cohen's *d* = 0.52, and intention to recommend the device to others facing sleep problems, *t*_(78)_ = 3.11, *p* < 0.005, Cohen's *d* = 0.69 (Table 2). Compared to the active control group (21.9%), significantly more participants in the experimental group (78.1%) reported that the SleepCogni device was more effective than other approaches or treatments they used in the past to improve their sleep, $\chi_{\left(2\right)}^{2}$ = 14.24, *p* = 0.001.

**Table 2:** Raw means with standard errors from the user experience questionnaire.

	**Group**			
	**Placebo**		**Experimental**	
**Measure**	**Mean**	**SE**	**Mean**	**SE**
Perceived usefulness	2.98	0.130	3.41	0.130
Perceived ease of use	4.39	0.113	4.40	0.113
Perceived ease to sleep	3.49	0.162	3.66	0.162
Intention to use in the future	6.52	0.471	7.60	0.471
Recommend to others	6.85	0.426	8.72	0.426
Overall satisfaction	6.54	0.448	7.61	0.611
Compared to other approaches or treatments	1.94	0.109	1.42	0.102

Compared to 3 participants (7.5%) in the control group who voluntarily left comments about their sleep in the Consensus Sleep Diary, 40% of participants (*n* = 16) in the experimental group did so, which was a statistically significant difference, $\chi_{\left(1\right)}^{2}$ = 9.94, *p* = 0.002. Content analysis indicated that participants using the SleepCogni device felt in control of their sleep, emotions, and environment; the device also helped them focus their thoughts and not ponder or worry about sleep and reduced the need to use other distractors to fall asleep (e.g., habitual use of the mobile phone).

## Discussion

The present study examined the effectiveness of SleepCogni, an active-feedback device, in improving sleep continuity outcomes in people with insomnia disorder (i.e., >3 months) using a parallel group randomized trial. It was hypothesized that participants in the experimental group would have lower scores regarding insomnia severity and report improved subjective continuity of sleep compared to participants in the control group. The results supported this hypothesis regarding the insomnia severity symptoms, with participants in the experimental group reporting significantly lower insomnia severity. In particular, the mean differences observed (−4.2 points in the ISI scores in our study) between the baseline and post-intervention measures in insomnia severity are equivalent to those reported in the extant literature (between −5.00 and −3.48 points on the ISI) about the effects of digitally delivered CBT-I on insomnia severity (see meta-analysis by Soh et al., 2020). A change of 6 points on the ISI is considered clinically meaningful (Yang et al., 2009), but the mean difference between groups did not reach this threshold; however, a substantial proportion of users reached this within the trial (37.5% in the experimental vs. 7.5% in the control arm, a 5-fold difference). Therefore, the SleepCogni intervention used in the present study appears to be a successful treatment for insomnia, especially considering improvements were seen within 1 week of treatment. However, although the rapid reduction in insomnia complaints is notable it could perhaps also be short-lasting. Our study only had one post-treatment assessment, and since the ISI focuses on past sleeping complaints, participants' responses reflected their treatment experience. As such, it is possible that complaints are only lowered while actively using the device. To disentangle this and investigate longer lasting benefits, an extended follow-up in future studies will be needed, in addition to validating the present findings in a larger and more representative clinical population. However, the present study does indicate that the SleepCogni device could usefully complement existing treatments for insomnia or as a monotherapy.

Furthermore, in support of the study's hypothesis, participants in the experimental group reported improvements in subjective sleep continuity, as indicated by the sleep diary data. Specifically, participants using the SleepCogni device displayed improved sleep efficiency and total sleep time compared to the control group (Table 1; Time × Treatment). However, it is notable that subjects made a number of errors in their sleep diary data that could be deduced from their sleep diary entries but also, interestingly, objectively from SleepCogni usage timestamps of button presses. For example, some users indicated that they started initiating sleep at a certain time, but timestamps of the SleepCogni device use indicated otherwise. This represents either a mismatch between the estimated and objectively recorded start time of their sleep window or that participants did not follow the instructions about using the device each night at their initial sleep initiation. Our preliminary comparison of sleep diary data with SleepCogni recordings indicates that subjective and objective measurements of the sleep initiation times are not always aligned, as reported in previous research (Lockley et al., 1999; Rezaie et al., 2018). It could be possible for the SleepCogni device to supply objective, user-inputted sleep diary data that is recorded automatically while using the device (Scott et al., 2023). In addition, SleepCogni could provide insights into when and how frequently users need assistance with sleep initiation and reinitiation. One specific use of such data would be in treating paradoxical insomnia, a condition in which a patient's insomnia complaints disagree with objective measures of their sleep (Rezaie et al., 2018). Data from SleepCogni usage could allow the CBT-I therapist to discuss another dimension of sleeping behavior during therapy, namely, sleep initiation and engagement with the treatment, as measured by the SleepCogni device through active input from the patient. Future work, including objective measures of sleep, would be valuable for directly studying the discrepancy between subjective and objective measures of sleep and insomnia complaints.

Finally, partially supporting the second hypothesis of the study, participants in the experimental group reported more positive user experience scores than participants in the control group, regarding the perceived usefulness of the device. Also, experimental group participants were significantly more likely to recommend the device to others (e.g., friends or relatives) facing sleep problems and perceived the SleepCogni device as more effective than other approaches or treatments they used in the past to improve their sleep quality and alleviate insomnia symptoms. Although these beliefs reflect subjective experiences, these findings provide initial evidence about the SleepCogni device's feasibility in improving the sleep experience of users. This is important considering that the devices used in the experimental and the control group were identical except for the absence of tactile stimuli in the control group and the button press. This also explains the non-significant differences in the perceived easiness of learning to use the device between the two groups—after all, the same use instructions were available to both groups. Regarding the qualitative user experience comments, 40% of participants (16 out of 40) in the experimental (active) group voluntarily commented on their experience, but only 7.5% (3 out of 40) of participants in the control (sham) group did so, and this difference was statistically significant, $\chi_{\left(1\right)}^{2}$ = 9.94, *p* = 0.002. Based on their comments, SleepCogni device users in the experimental group felt more in control of their sleep, emotions, and environment; experienced less mind wandering and more distraction from unhelpful sleep-related thoughts; and engaged less in counterproductive behaviors, such as using their mobile phones to fall asleep.

This study has certain limitations. First, the trial employed a rather short timeframe (i.e., 1 week) to determine the effectiveness of using the SleepCogni device, lacking follow-up measures for long-term effectiveness (e.g., at 3 or 6 months). Future research may examine the long-term effects of using the SleepCogni device on sleep-related outcomes. Second, we did not have any pretrial data from sleep diaries, which meant we could not do a randomization check for this variable in the study and, hence, our results on sleep diary records (Table 1) could reflect preexisting differences in this measure between treatment arms. Third, this study tested the efficacy and user experience of SleepCogni in subjects without a range of possible confounding comorbidities and subjects were relatively young. Within the population of insomnia sufferers, comorbidities are common, and therefore real-life testing should focus on any interacting comorbidities that may enhance or reduce responses to treatment. Fourth, subjects in the placebo group may have somehow realized they were in the control arm of the study. However, this is unlikely as the placebo group showed an improvement in their ISI scores and the user experience data did not include any suggestion that subjects were aware of the placebo condition. Fifth, the study used subjective measures of insomnia complaints. Relevant objective measures of behavior and sleep include actigraphy and/or polysomnography. Note, however, that objective measures of insomnia are not always aligned with subjective measures of insomnia (Lockley et al., 1999; Rezaie et al., 2018). Still, the reliance on subjective measures of insomnia alone, even though forced by study limitations including implementation in the home setting, is a weakness of the study.

Notwithstanding these limitations, the present study is the first to demonstrate the effectiveness of an active-feedback device (SleepCogni) in reducing insomnia symptom severity and improving subjective sleep continuity. Note that for insomnia severity (measured using the ISI), we did have baseline measurements, and groups did not differ at baseline. The results from the user experience analysis further indicate that the handheld device used in this study was, to a large part, widely accepted by end users. Most importantly, the present study highlights the importance, usefulness, and relevance of a handheld product (e.g., SleepCogni device) that empowers individuals to self-manage their symptoms. Such handheld products may be used as a first-line response treatment option to support patients in self-managing (and alleviating) their symptoms while waiting to receive full clinical treatment (e.g., CBT-I). This is an important aspect considering the current backlog of healthcare services across different countries resulting from the COVID-19 pandemic's impact on access to healthcare services (Tille and Zapata, 2022).

The clinical implications of the present study suggest that the SleepCogni device could be used either as a monotherapy or as a complement to existing treatments for insomnia, providing an effective self-management tool while individuals await full clinical treatments such as CBT-I. Future research could examine the synergistic effects of combining the SleepCogni device with CBT-I. While CBT-I equips patients with insomnia to regulate their symptoms and their sleep (i.e., offline symptom management), the SleepCogni device allows patients to self-manage their symptoms in real time, potentially contributing to improved sleep efficiency (i.e., online symptom management). Furthermore, the SleepCogni device can assist clinicians by offering an objective assessment of their patients' difficulties with sleep initiating or reinitiating. SleepCogni provides unique insights into sleep struggles in the home environment, through recorded active user input and by tracking improvements in sleep and device use over time.

## Data Availability

The raw data supporting the conclusions of this article will be made available by the authors, without undue reservation.
