# Supplementary material for: Novel active-feedback device improves sleep quality in insomnia disorder: a randomized placebo-controlled trial
Source: Front Sleep. 2025 May 16;4:1452213. doi: 10.3389/frsle.2025.1452213 (PMC12713996; doi:10.3389/frsle.2025.1452213)
Supplement: Supplementary file 1 [file Data_Sheet_1.pdf]

CONSORT diagram showing the flow of participants through the trial described in "Novel Active-Feedback Device improves sleep quality in Insomnia Disorder: a randomized placebo-controlled trial"

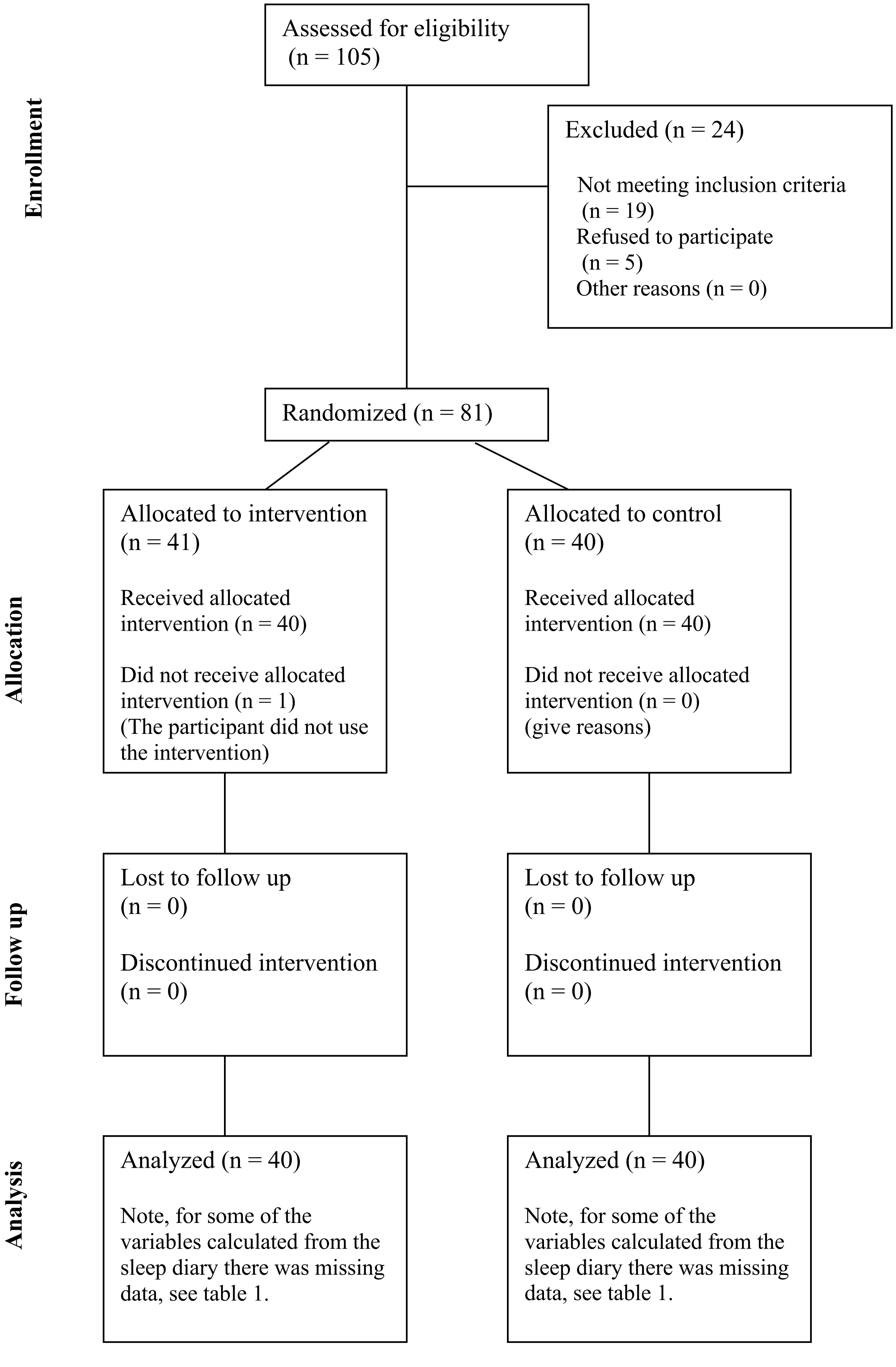

User Experience Questionnaire

Below you may find a list of User Experience questions that tap key factors used in most technology acceptance/user experience studies. The questions are bespoke to reflect the use and scope of the specific technological application/device. The factors (and the relevant annotations in the questionnaire) appear as follows - this is not an exhaustive list and we may add/modify the factors or specific questions according to the needs of the study.

SRU = Self-reported Use

PU = Perceived usefulness of the device

PEOU = Perceived ease of using the device

BI = Behavioural Intention

SAT = Satisfaction

END = Endorsement of the device

SRU1. How often did you use (name of device) in the last week?

□ Not at all □ 1-2 times a week □ 1-2 times a day □ More than 1-2 times a day

SRU2. If you DID NOT USE (the device) AT ALL last week what were the main reasons? (Please tick as many as apply)

□ It disrupted my sleep routine

□ It did not find any use of it

□ It interfered with my effort to fall asleep

□ It made me feel anxious and distressed

Other (please explain): ……………………………………………………………………………………….

YOUR IMPRESSIONS ABOUT USING THE (DEVICE)

To be completed only if you used the device for at least two days/nights in the last week

How much do you agree or disagree with the following statements?

**Using the device…**	**Strongly Disagree**	**Disagree**	**Neither agree, nor disagree**	**Agree**	**Strongly Agree**
PU1 …improved my effort to fall asleep	1	2	3	4	5
PU2 …helped me fall asleep more easily	1	2	3	4	5
PU3 …reduced my worries over falling asleep	1	2	3	4	5
PU4 …helped me control my sleep routine	1	2	3	4	5
PU5 …helped me relax	1	2	3	4	5
PU6 …helped me control my thoughts more easily	1	2	3	4	5
PU7 …helped me control my emotions	1	2	3	4	5
PEOU1 Overall, I found it easy to use the device	1	2	3	4	5
PEOU2 Learning to use the device was easy and understandable	1	2	3	4	5
PEOU3 It was easy to fall asleep with the device	1	2	3	4	5
PEOU4 Overall, I did not face any difficulties using the device at the end of the week	1	2	3	4	5

SAT1. Overall, how satisfied are you <u>with using the device</u>? Please circle the answer that best represents how you feel.

Extremely dissatisfied										Extremely satisfied
0	1	2	3	4	5	6	7	8	9	10

SAT2. Overall, how much did the device help you to fall asleep? Please circle the answer that best represents how you feel.

Not at all										Very much
0	1	2	3	4	5	6	7	8	9	10

SAT3. Overall, how confident do you feel to successfully initiate sleep using the device? Please circle the answer that best represents how you feel.

Not at all										Very much
0	1	2	3	4	5	6	7	8	9	10

BI1. If you had the opportunity, how likely would you be to use the device in the future? Please circle the answer that best represents how you feel.

Not at all										Very much
0	1	2	3	4	5	6	7	8	9	10

END1. Would you recommend the device to a friend or relative of yours who faced sleep problems? Please circle the answer that best represents how you feel.

Definitely not										Definitely yes
0	1	2	3	4	5	6	7	8	9	10

Compared to other approaches or treatments you used before, how would you rate this device? Please select only the answer that best represents how you feel.

- ☐ It was <u>more effective</u> than other approaches or treatments I used before
- ☐ It was <u>equally effective</u> to other approaches or treatments I used before
- ☐ It was <u>less effective</u> than other approaches or treatments I used before

Table S1. Pre-treatment values of the measures for the control and experimental group. The self-reported data are presented in the result section as well but summarized here for convenience.

**Measure**	**Pre-treatment for control group**	**Pre-treatment for experimental group**
ISI	*M* = 16.50, *SD* = 3.62	*M* = 16.75, *SD* = 4.54
Anxiety	M = 7.43, SD =3.74	M = 5.80, SD = 3.85
Depression	M = 11.38, SD = 3.37	M = 10.10, SD = 4.47
Age	M = 24.4, SD = 8.89; range 18-60	M = 29.1, SD = 13.7; range 18-71
Sex	33 female, 7 male	32 female, 8 male
